# Roles of endocrine FGF signaling in metabolic health and cancer

**DOI:** 10.1186/2049-3002-2-S1-P43

**Published:** 2014-05-28

**Authors:** Yongde Luo, James Abbruzzese, Wallace McKeehan

**Affiliations:** 1 Center for Cancer and Stem Cell Biology, Institute of Biosciences and Technology, Texas A&M University Health Science Center, Houston, TX, USA; 2 Division of Medical Oncology, Duke Cancer Institute, Duke University, Durham, NC, USA; 3 Department of Medicine, Wenzhou Medical University, Wenzhou, Zhejiang, China

## 

Endocrine FGF21, FGF19 and co-receptor betaKlotho divert the roles of the canonic fibroblast growth factor receptors (FGFRs) complex from maintaining growth/proliferation homeostasis to local and systemic metabolic homeostasis. These endocrine factors target adipocytes and hepatocytes leading to alleviation of obesity, fatty liver disease and metabolic abnormalities which are contributing factors to the progression of several types of tumor. Here we determined whether systemic and microenvironmental metabolic alterations caused by FGFR4 deficiency affect tumorigenesis in breast where FGFR4 is negligible in contrast to FGFR1. Breast tumors were induced by overexpression of TGFα that activates Her2 in ductal and lobular epithelium surrounded by adipocytes. Mammary tumorigenesis and alterations in systemic and breast microenvironmental metabolic parameters and regulatory pathways were analyzed.

The ablation of FGFR4 had no effect on cellular homeostasis and Her2 activity of normal breast tissue; however, it reduced TGFα-driven breast tumor incidence and progression and improved host survival. Notable increases in hepatic and serum FGF21, adiponectin and adipsin, and decreases in systemic Fetuin A, IGF-1, IGFBP-1, RBP4 and TIMP1 were observed. These alterations affected adipogenesis and secretory function of adipocytes as well as lipogenesis, glycolysis and energy homeostasis associated with the functions of mitochondria, ER and peroxisomes in the breast and tumor foci. The ablation also affected several pro-inflammatory factors. In line with these data, the adipocyte-specific FGFR1 deficiency that disrupts FGF21 signaling elevated adipose ER stress response, and modulated the syntheses of phospholipids and lipid mediators and lipid droplet expansion. In contrast, overexpression of FGF21 enhances lipolysis, mitochondrial fatty acid oxidation and energy uncoupling, and subdues ER stress, pro-inflammatory and oxidative stress that maintains adipose and metabolic health. Treatment with an inhibitor of NAMPT involved in the pathways inhibited the growth and survival of breast tumor cells and tumor-initiating cell-containing spheres.

Although the primary role of FGFR4 in metabolism occurs in hepatocytes, its ablation results in a net inhibitory effect on mammary tumor progression, suggesting that the tumor-delaying effect of FGFR4 deficiency may be largely due to elevated anti-obesogenic FGF21 that triggers stress-reducing and tumor-suppressing signals from both peripheral and breast adipocytes. The predominant changes in metabolic pathways suggested roles of metabolic effects from both peripheral and breast adipocytes on metabolic reprogramming in breast epithelial cells that contribute to the suppression of tumor progression. These results provide new insights into the contribution of systemic and microenvironmental metabolic effects controlled by endocrine FGF signaling to breast carcinogenesis.

